# Vitamin E and Bone Structural Changes: An Evidence-Based Review

**DOI:** 10.1155/2012/250584

**Published:** 2012-10-17

**Authors:** Isa Naina Mohamed, Boekhtiar Borhanuddin, Ahmad Nazrun Shuid, Nur Farhana Mohd Fozi

**Affiliations:** Pharmacoepidemiology and Drug Safety Unit, Department of Pharmacology, Faculty of Medicine, Universiti Kebangsaan Malaysia (The National University of Malaysia), Jalan Raja Muda Abdul Aziz, 50300 Kuala Lumpur, Malaysia

## Abstract

*Purpose*. This paper explores the effects of vitamin E on bone structural changes. *Methods*. A systematic review of the literature was conducted to identify relevant studies about vitamin E and osteoporosis/bone structural changes. A comprehensive search in Medline and CINAHL for relevant studies published between the years 1946 and 2012 was conducted. The main inclusion criteria were published in English, studies had to report the association or effect of vitamin E and osteoporosis-related bone changes, and the osteoporosis-related bone changes should be related to lifestyle variables, aging, or experimentally-induced conditions. *Results*. The literature search identified 561 potentially relevant articles, whereby 11 studies met the inclusion criteria. There were three human epidemiological studies and eight animal experimental studies included in this paper. Four animal studies reported positive bone structural changes with vitamin E supplementation. The rest of the studies had negative changes or no effect. Studies with positive changes reported better effects with tocotrienol vitamin E isomer supplementation. *Conclusions*. This evidence-based review underscores the potential of vitamin E being used for osteoporosis. The effect of one of the vitamin E isomers, tocotrienols, on bone structural changes warrants further exploration. Controlled human observational studies should be conducted to provide stronger evidence.

## 1. Introduction

Osteoporosis is a progressive medical condition in which bone density slowly decreases with advancing age. Osteoporosis is often called “silent” because bone loss occurs without symptoms. An estimated 10 million men and women in the United States have osteoporosis and a further 34 million are at risk [1]. Bone is a highly vascularized connective tissue that contains haematopoietic bone marrow, calcium, and phosphate [2]. Calcium is the most abundant mineral found in bone with approximately 98% of human body calcium stored in bone. There are two principal cell types in bone: osteoclast and osteoblast. A balance between bone formation by osteoblasts and bone resorption by osteoclasts is needed for bone remodeling and bone development [3]. Osteoporosis is defined as a bone mineral density (BMD) that lies 2.5 standard deviations or more below the average value for young healthy women. Osteoporosis is a progressive systematic skeletal disorder characterized by low bone mineral density (BMD), deterioration of microarchitecture of bone tissue, and susceptibility to fracture caused by bone resorption [4, 5]. Osteoclast cells have the ability to release free radicals, such as reactive oxygen species (ROS) that will destroy calcified bone tissue and therefore play an integral part in bone remodeling when coupled with osteoblast cells [6–10]. Recent epidemiological studies have also indicated a relationship between oxidative stress and osteoporosis [11, 12].

Vitamin E occurs naturally in eight isoforms: *α*, *β*, *γ*, and *δ* isomers of tocopherols and tocotrienols. Each isomer of vitamin E consists of an aromatic chromanol ring and a side chain. Tocotrienols possess an unsaturated farnesyl (isoprenoid) side chain compared to tocopherols, which have a saturated phytyl side chain [13]. The unsaturated side chains of tocotrienols allow them to penetrate more efficiently into the membrane lipid bilayer. Vitamin E has good antioxidant activity, which varies among the different isomers [14]. Vitamin E is a strong antioxidant that plays a vital role in the endogenous defense against peroxidation of membrane lipids [15]. Tocopherols are abundant in polyunsaturated vegetable oils and in the germ of cereal seed [16]. Tocotrienols are abundant in palm oil, cereal grains, and rice bran [17]. Commercial availability of vitamin E is mostly in the form of *α*-tocopherol, which is taken as an antioxidant supplement [18]. *α*-Tocopherol has the highest biological activity and is the most abundant form of vitamin E in human tissues and serum [19, 20] as it is selectively retained in the body [21, 22]. Palm olein (refined, deodorized, and bleached palm cooking oil) contains 196 ppm *α*-tocopherol, 201 ppm *α*-tocotrienol, 372 ppm *β*-tocotrienol, and 96 ppm *γ*-tocotrienol [23]. As described, tocotrienols are found abundant in palm oil and have been reported to be better antioxidants compared to tocopherols [24, 25].

Evidence of vitamin E having a beneficial effect against osteoporosis has been actively researched only recently. Vitamin E has been reported to play a role in increasing bone density. According to researcher NS Ahmad and colleagues in their research on nicotine-treated rats, vitamin E increases the trabecular bone (a spongy-looking bone), prevents bone calcium loss by neutralizing antioxidants, and decreases bone loss of calcium in rats without ovaries [26]. Vitamin E supplementation was able to protect bones from oxidative damage by scavenging free radicals [27, 28] and was able to maintain bone matrix trophysm and stimulate trabecular bone formation [29, 30]. Previous studies reported that vitamin E supplementation protects against bone loss and damage caused by oxidative stress, which is induced by sex hormones deficiency [31, 32] or oxygen-derived free radicals [33–35]. Postovariectomised rats have similar bone changes to those of postmenopausal women [36]. Vitamin E supplementation protects against bone loss and restores bone strength in the aged mouse [37] and ovariectomized rat [32, 38]— both are accepted osteopenic models.

Nicotine has been reported to increase proinflammatory mediators (through oxidative stress), resulting in bone loss and reduced bone mechanical strength (through inhibition of osteoblasts) in rats [39–41]. In humans, smoking is a recognized risk factor for osteoporosis [42, 43]. Recent studies utilising nicotine-induced rat model have reported that vitamin E was able to prevent the increment of bone-resorbing cytokines [26] and reverse the damage on bone histomorphometry [44]. 

Calcium plays an integral part in bone metabolism and remodeling [45]. A vitamin E-deficient diet will result in bone damage, probably due to impaired calcium absorption [46, 47] that leads to a state of calcium deficiency [48] and increased free radical activity [49]. 

Elevated levels of bone-resorbing cytokines, mainly interleukin 1 and 6 (IL-1 and IL-6), are known to be associated with accelerated bone resorption after menopause [50–52]. IL-1 is secreted by monocytes in an estrogen deficiency state that will induce osteoblast to secrete IL-6 [50]. IL-6 will stimulate osteoclast proliferation and subsequently increase bone resorption [52, 53]. Vitamin E, especially tocotrienol that was reported to be more potent compared to tocopherol, was reported to be able to prevent the rise of serum IL-1 and adverse effects of free radicals on trabecular bone structure [33, 34]. 

The increasing number of studies, especially in the past decade, which focused on the role of vitamin E in the prevention or treatment for osteoporosis warrants a review. The aim of this evidence-based review is therefore to explore original research articles in order to determine the effects of vitamin E on bone structural changes. 

## 2. Methods

### 2.1. Literature Review

A systematic review of the literature was conducted to identify relevant studies about vitamin E and osteoporosis/bone structural changes. To conduct a comprehensive search of health science journals, we used Medline via Ovid Medline (published between 1946 and March 2012) and CINAHL via Ebscohost (published between 1946 and 2012). The search strategy involved a combination of the following two sets of key words (1) vitamin e OR vit* e OR tocotrienol OR tocopherol; (2) bone* OR bone metabolis* OR bone mineral* OR osteoblast* OR osteoclast* OR osteoporo* OR osteopen* OR osteogen*. 

### 2.2. Selection of Research Articles

The results were limited to studies that were published in English language that included abstracts. To be included, studies had to (1) report the association or effect of vitamin E and osteoporosis-related bone changes and (2) the osteoporosis-related bone changes should be related to lifestyle variables, aging, or experimentally-induced conditions. Papers were excluded if the studies were related to (1) osteoporosis that is related to other pathological changes; (2) reviews, news, letter, editorials, or case studies; (3) bone fracture healing; or (4) fetal bone or bone marrow formation. 

### 2.3. Data Extraction and Management

We selected papers to be included in the review in three phases. First, we excluded any paper that did not match the inclusion criteria based solely on the title. Second, we screened all the abstracts of the remaining papers and then excluded a second group of papers that did not meet our inclusion criteria. Lastly, we read the remaining papers from the second phase to exclude any paper that did not meet our inclusion criteria. 

After the initial screening of the titles and abstracts, duplicates were removed and the remaining papers were again screened by at least two reviewers. The inclusion of the full papers into the review had to be agreed by at least two reviewers before the data extraction phase. Any discrepancies were resolved through discussion between the reviewers. Data extraction was performed independently and in a standardized manner with the use of a data collection form. We recorded the following data from the studies: (1) the type of study and vitamin E analog studied; (2) a brief description of the sample/population of the study; (3) a brief description of the methods used in the study; (4) the brief description of the results of the study; (5) our comments and conclusion of the study.

## 3. Results

### 3.1. Search Results

The literature searches identified 561 potentially relevant articles. Two reviewers independently assessed all articles for inclusion or exclusion based on the title and abstract. A total of 42 articles were retrieved for further assessment and data extraction. Fifteen of these articles were excluded because they did not focus on primary studies (*n* = 8) or because they were not related with vitamin E and osteoporosis. 

**Figure 1:**
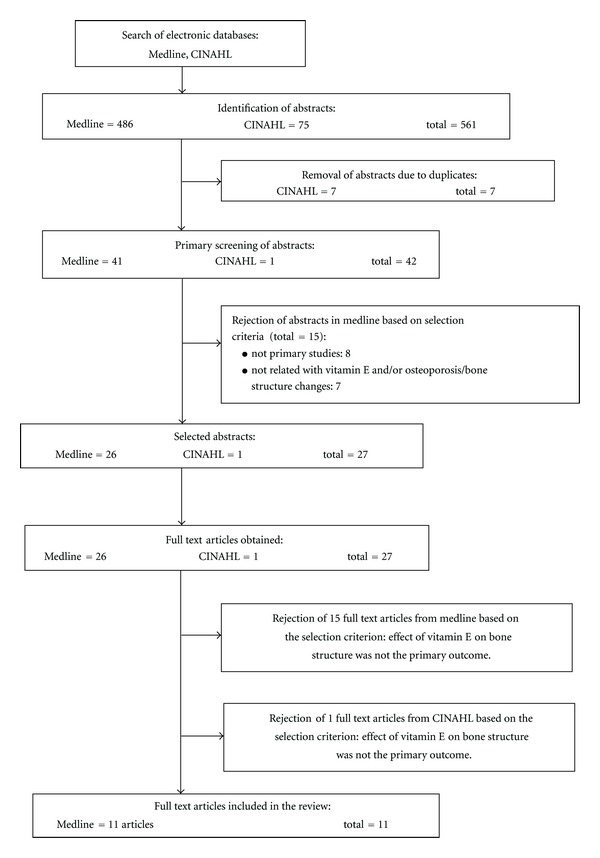
Flow chart to show the selection process of the articles in this review.

Differences of opinion between the reviewers regarding the inclusion or exclusion of the full articles were resolved by discussion. 16 articles from the remaining 27 articles were excluded based on the inclusion and exclusion criteria. A total of 11 articles were included for the purpose of this review. A flow chart of the selection and paper process, including reasons for exclusion, is shown in Figure 1. 

### 3.2. Study Characteristics

The summary of the characteristics of all studies is displayed in Table 1 (animal studies) and Table 2 (human studies). All studies were conducted after the year 2000 with the majority conducted in the past five years. There were three human studies and eight animal studies that used rats as their study model. Although only three of human studies were included in this paper, each of these studies by Macdonald et al. [60], Maggio et al. [59], and Wolf et al. [61] had a large population sample size of at least 150 women. The study by Wolf et al. [61] had a sample of 11,068 participants and hence provided ample power and confidence for the generated results. All human studies involved women only, with bone mineral density (BMD) measurement being the main measured outcome, thus providing a highly reliable indicator for osteoporosis. Studies by Macdonald HM and Wolf RL collected information on vitamin E consumption through the use of questionnaires. It was not mentioned whether the questionnaires were validated. Vitamin E consumption from diet and supplements were then correlated with BMD measurements to determine the effect of vitamin E consumption on BMD. Maggio compared the plasma vitamin E level of osteoporotic participants versus nonosteoporotic participants to determine if there are any differences in vitamin E levels of women suffering from osteoporosis. 

One animal study used Winstar rats with the remaining seven studies using Sprague-Dawley rats. Rats were of various ages, with the number of rats for each study (ranging from 24 to 96 rats) kept to a minimal number due to animal ethics requirements. Seven studies [32, 34, 44, 54, 55, 57, 58] assessed trabecular and cortical bone structure using bone histomorphometry analysis of the rat bones after treatment with vitamin E. Three studies [32, 55, 56] conducted various blood biochemical tests, three studies [32, 54, 56] conducted BMD scans, one study [57] conducted a biomechanical test to assess bone strength, and one study [56] used Micro-CT to assess trabecular and cortical bone structure. All animal studies were conducted using experimental design which compared the outcome of vitamin E treated groups (tocopherol, tocotrienol, or combination of various amounts) with control or sham group. 

## 4. Effects of Vitamin E on Osteoporosis for Human Epidemiological Study

There were three human studies included in this paper. The study by Maggio et al. [59], was conducted to determine whether antioxidant defense (including vitamin E) decreased in osteoporotic elderly women by measuring antioxidant plasma levels of participants. There were a total of 150 women participants, whereby 75 women with diagnosed osteoporosis were compared with 75 women with normal BMD (control). The sample characteristics were as follows:mean age: 70.4 years; mean BMI: 25.3; mean years since menopause: 22.8 years. The reported results indicated that the mean plasma levels of vitamin E were significantly lower in osteoporotic women, if compared to control participants (*P* < 0.001). The authors suggested the need of further research to determine the relevance and the mechanism of action between low levels of vitamin E and osteoporosis. 

Macdonald et al. [60], conducted a large longitudinal study with 891 women participants to determine the association between antioxidants (including vitamin E) intake and BMD. The characteristics of study participants were as follows: mean age: 53.9 years; mean BMI: 26.1; total vitamin E intake: 13.3 mg. Macdonald concluded that there was no evidence of any association between nutrient intake and BMD change. Total vitamin E intake (dietary plus supplementation) correlated positively with BMD, but was not significant. Dietary vitamin E intake was significantly correlated with BMD (*P* < 0.01), but was negatively correlated. 

The largest human epidemiological study included in this review was conducted by Wolf et al. [61], involving 11,068 women participants (mean age: 63.2 years; mean BMI: 28.3; mean total vitamin E intake: 28.9 mg). Due to the high number of participants, the regression analysis done in this study had significant power, with multiple covariate adjustments performed. Wolf reported positive correlation between dietary vitamin E and femoral neck BMD when adjusted for age only. After adjusting for multiple BMD related covariates, no significant association was found between vitamin E consumption and all BMD parameters.

## 5. Effects of Vitamin E on Osteoporosis for Human Animal Study

A total of 10 animal studies were included in this paper. Ahmed et al. have two studies included in this paper. Shuid et al. used bone histomorphometry and biomechanical strength model, whereby *γ*-tocotrienol supplementation produced greater trabecular volume and number compared to control rats and rats supplemented with *α*-tocopherol [57]. Using Ferric nitrilotriacetate (FeNTA) to induce diabetes in rats and subsequently bone damage, N.S. Ahmad et al. reported that palm tocotrienol mixture (consisting of *α*-TT 30.7%, *γ*-TT 55.2%, *δ*-TT 14.1%) supplementation was able to prevent bone damage [34]. Palm tocotrienol was also reported to have superior protection properties compared to *α*-tocopherol.

 Ima-Nirwana and Suhaniza reported that supplementation with *γ*-tocotrienol or palm vitamin E successfully prevented osteoporosis in adrenalectomised rats replaced with dexamethasone, if compared to control and rats supplemented with *α*-tocopherol [54]. Norazlina et al. in their study (published in the year 2000) compared ovariectomized rats and nonovariectomized rats supplemented with palm vitamin E or *α*-tocopherol. There was no significant change between the treatment groups, in terms of BMD obtained from histomorphometry [32]. Hermizi et al., in a 2009 study using nicotine-induced rat bone damage model, reported that vitamin E was able to reverse the effect of nicotine damage on bone structure and noted *γ*-tocotrienol supplementation caused significantly higher mineral appositional rate and bone formation rate, if compared to tocotrienol-enhanced fraction and *α*-tocopherol treated-group [44].

In a recent study by Mehat et al. 2010, rats supplemented with oral vitamin E for four months had increased trabecular and osteoid bone volume, and reduction of bone resorption parameters [58]. *γ*-tocotrienol supplementation produced the best bone measurements improvement, if compared to *δ*-tocotrienol and *α*-tocopherol [58]. Smith et al. reported that *α*-tocopherol did not have any effects on bone histomorphometry parameters when supplemented at varying doses in hindlimb unloaded rats, if compared to ambulatory rats [55]. Chai et al. reported similar negative findings on varying doses of *α*-tocopherol supplementation in male orchidectomized rats [56]. No significant BMD parameters were observed and Chai et al. concluded that *α*-tocopherol supplementation was not able to reverse bone loss due to gonadal hormone deficiency [56].

## 6. Discussion

This paper has mixed main findings. There was strong evidence of the benefit of vitamin E supplementation in rats, whereby positive changes in bone structure were demonstrated for four of the eight animal studies [34, 44, 57, 58]. Three out of the four remaining animal studies [32, 54, 57] reported positive findings of vitamin E supplementation for biochemical results, although not for bone structure parameters. Only Chai et al. reported no positive finding for vitamin E supplementation in rats. Rat animal model has become acceptable for human bone studies due to the similar mechanism of control gain and loss of bone mass compared to humans. All the three human studies reported no association between vitamin E consumption and BMD changes [59–61]. 

Nicotine causes an increase of proinflammatory mediators (oxidative stress) that results in bone loss and reduction of bone mechanical strength (through the inhibition of osteoblasts) in rats [39–41]. In humans, smoking is a recognized risk factor for osteoporosis [42, 43]. Hermizi et al. reported that vitamin E treated rats were able to recover from nicotine-induced bone damage, where tocotrienol mixture was more potent compared to tocopherol [44]. Postulated mechanisms were antioxidative effects of vitamin E and the reduction of free radicals.

Five studies denoted a differing effect towards bone structure between vitamin E isomers, mainly between tocotrienol and tocopherol [34, 44, 54, 57, 58]. It has been postulated that tocotrienol has better activity compared to *α*-tocopherol due to the higher mobility of polyenoic lipids of tocotrienol in the membrane bilayer and therefore is more mobile and less restricted in its interaction with lipid radicals in the membranes [24]. Both of Ahmad et al. studies employed histomorphometry technique to view changes of rat bone structures [34, 57]. In their 2005 study, vitamin E treated rats were able to maintain their bone structure, resisting FeNTA-induced bone damage. Ahmad et al. supplemented *α*-tocopherol and tocotrienol mixture on different groups of rats and noted that only rats treated with tocotrienol mixture was able to effectively resist bone damage induced by FeNTA [34]. In a recent study by shuid et al. (2010), they reported that vitamin E supplementation had positive anabolic changes on bone structure (i.e., increased trabecular volume and number) compared to control rats [57]. Both studies concluded that improvement was superior among tocotrienol treated-rats compared to *α*-tocopherol-treated rats. Mehat et al. conducted a study specifically to determine bone structural changes of rats supplemented with different isomers of vitamin E (*α*-tocopherol versus *δ*-tocotrienol versus *γ*-tocotrienol) [58]. Bone histomorphometry analysis revealed that all isoforms promote bone growth in rats with *γ*-tocotrienol, producing the best effect in both static and dynamic measurements in bones. Mehat et al. did not suggest a mechanism or reason to explain the differences observed between the isomers of vitamin E.

Three out of four remaining animal studies reported positive results for positive biochemical changes, but without any significant bone structural changes. Among the biochemical changes reported were the increase of bone calcium content [32, 54], the reduction in oxidative status measured using plasma ferric-reducing ability [55], the improvement of serum alkaline phosphatase (ALP) and the reduction of serum tartrate-resistant acid phosphatase (TRAP) [32], and the improvement of whole body fat mass [54]. Serum ALP is a measurement of osteoblastic activity, while serum TRAP is specific for osteoclastic activity [62]. Ima-Nirwana and Suhaniza reported *γ*-tocotrienol-treated rats had the best effect on body composition and postulated that the effect could be due to tocotrienol's antioxidant effect or improvement of calcium transport and utilization [54]. Improvements of either bone structure parameters or biochemical parameters were mostly attributed to vitamin E through its antioxidant effects, free-radical scavenging capability, protection from cellular lipid peroxidation, improvement of calcium transport and utilization, and the suppression of bone resorbing cytokines, IL-1 and IL-6. 

Maggio et al. in his study confirmed that plasma concentration of vitamin E is lower in osteoporotic women when compared to nonosteoporotic women [59]. However, all the three human epidemiological studies in this paper reported no significant association between vitamin E consumption and BMD improvement [59–61]. The largest study, conducted by Wolf et al. with 11,068 participants, had positive age-adjusted regression analysis for dietary Vitamin E and femoral neck BMD (*P* = 0.002) association, but negative association for total vitamin E (*P* < 0.0001) [61]. Due to the large number of participants and sufficient statistical power, Wolf et al. were able to perform regression analysis that adjusted for more than 50 important BMD-related covariates. However, no significant association was reported. Wolf et al. offered several possible explanations of the mixed results: the variety in exposure measurement, outcome measurement and BMD site measurement, and other confounding factors. Among the three human epidemiological reviews, Macdonald et al. had reported negative correlation between dietary vitamin E and BMD measurements (*P* < 0.01), although this association was not persistent for the total vitamin E consumption, suggesting a possible opposite outcome if vitamin E was taken in larger doses [60]. 

Comparing human epidemiological studies with animal studies outcome is often difficult, as translation of results from animals to humans is hampered by various differences. The mechanisms of which vitamin E exerts its effects could be different in humans. Controlling environmental factors, food intake, and confounding factors is often possible in animal studies, but impossible in human epidemiological studies. Interactions between vitamin E and other antioxidants, nutrients, chemicals, and other food sources have to be explored. The stark differences, with almost opposite outcomes between animal studies and human epidemiological studies in this review, are therefore not surprising. All human epidemiological studies in this review reported consumption of vitamin E as a study variable, but the distinction between the different vitamin E isomers (tocopherols and tocotrienols) was not made. The majority of animal studies actually reported a positive outcome, or at least a superior effect for tocotrienols compared to tocopherols. This could offer a possible explanation for the discrepancy of the study outcomes. We were unable to provide a definitive answer whether vitamin E has any significant effects on bone structure, either positive or negative. The outcomes of studies included in this review were mixed. Half of the animal studies had positive changes on bone structure, whereas none of the human epidemiological studies had positive effects. Tocotrienols seemed to exert superior effects on bone structure and further studies concentrating on this isomer would need to be explored. Empirical human case-control studies or randomized control trials with vitamin E or its isomer derivatives would help ascertain the effect of vitamin E on bone structure.

**Strength and Limitation of This Review**

The research on the effects of vitamin E on osteoporosis is a promising field with important findings published in the last decade and a critical review is therefore highly relevant. Our search identified 11 research articles that were included in this paper and we believe that this is the first critical review on this subject matter that focuses on vitamin E and bone structural changes. We have also included both animal studies and human studies in this review, providing a better overview of the most recent and reliable evidence available.

This review has several limitations. Many studies did not differentiate the many isomers of vitamin E in their study. Due to the difference in the effect and activity of the various isomers, generalization of results and outcomes for the effect of vitamin E had to be further scrutinized. Many of the original research articles included in this review had other parameters included, especially blood biochemical parameters. The human studies focused on all antioxidants and mostly concentrating on vitamin C, instead of vitamin E. Nevertheless, results were thoroughly screened to avoid misrepresentation of the results of other antioxidants. All the human studies included in the review were epidemiological in nature, which presents with its own inherent weaknesses. Despite this limitation, the number of participants for each human epidemiological study was large. For example, the study by Wolf et al. had over 11,000 participants, providing ample statistical power and the inclusion of various adjusted covariates in the multiple-regression analysis.

**Recommendations**

Based on the heterogeneity of the study methods, especially among the animal studies, it is crucial that future studies use a standardized protocol to examine the effect of vitamin E various isomers on a consensual gold-standard parameter that assesses the osteoporotic state. Besides that, more effort should be made in designing controlled human observational studies that will help in reducing the numbers of potential confounders in the analysis of the result. These measures will ensure that proper meta-analysis could be conducted in the future to give us a clearer picture about the actual effect of vitamin E on osteoporosis.

## 7. Conclusion

This evidence-based review underscores the potential of vitamin E being used for osteoporosis, and sought to look into vitamin E and bone structure changes. The effect of vitamin E isomers, especially tocotrienols, on bone structural changes warrants further exploration. Additionally, controlled human observational studies should be conducted to provide stronger evidence. Due to the mix outcomes of the studies included in this paper, it is currently premature to state that vitamin E has positive, negative, or no effect on bone structure, at least until more studies are conducted.

## Figures and Tables

**Table 1:** Characteristics of animal studies included in the review.

STUDY	Type of study/vit E analog	Sample/ population	Methodology	Results (vitamin E and osteoporosis only)	Comments or outcomes
Norazlina et al. 2000 [32]	Animal study Bone mineral density *α*-Tocopherol Tocotrienol	3-month-old female Sprague-Dawley rats (*n* = 80) Half of rat were ovariectomised and half were left intact Rats were assigned into four group (10 rats for each groups) Duration: 10-month treatment	Three of the four groups were supplemented with palm vitamin E 30 mg/kg (PVE30), palm vitamin E 60 mg/kg (PVE60), or *α*-tocopherol 30mg/kg (ATF). The other group was supplemented with normal rat chow (RC). After 8 months of treatment, rats were sacrificed. The left femur and lumbar vertebra were dissected out and cleansed of all soft tissue for bone histomorphometry measurements: (a) bone mineral density measurements of the left femur and vertebra were obtained using the Dual-Energy A-ray Absorptiometer (b) bone calcium content (c) serum biomarkers of bone metabolism Serum alkaline phosphatase and serum tartrate resistant acid phosphatase were assayed and measured using a spectrophotometer at 405 nm.	(a) Bone mineral density Bone mineral density did not show any significant difference between all treatment groups. Palm vitamin E 30 mg/kg group of intact rats had higher bone mineral density in the distal part of femur compared to the ovariectomised. (b) Bone calcium content Both intact and ovariectomised rats supplemented with palm vitamin E 30 mg/kg weight rat had lower bone calcium content in femoral and vertebral bone. However, PVE60 and ATF groups were able to maintain bone calcium content. (c) Bone biomarkers The activity of ALP enzyme did not differ between all treatment groups for both intact and ovariectomised rats. The ALP enzyme of ovariectomised rats for group PVE30 and ATF was higher than intact control. The TRAP enzyme activity, PVE60 supplemented rat, was significantly lower compared to intact ATF group. The enzyme activity is significantly lower in ATF ovariectomised group, if compared to intact group.	Both tocopherol and tocotrienol act on bone, but in different mechanism of action. Alpha tocopherol reduced the activity of TRAP that maintains bone mineral density.

Ima-Nirwanaet and Suhaniza 2004 [54]	Animal study Histomor-phometry analysis *α*-Tocopherol *γ*-Tocotrienol	4-months-old male Sprague-Dawley rats (*n* = 42) Rats were adrenalectomized after two-day receipt Rats were randomly assigned into six groups. 7 rats for each group Duration: 8-week treatment	Rats randomly divided into six group as follows: (a) Group A—dexamethasone 120 *μ*g/kg + vehicle olive oil (b) Group B—dexamethasone 240 *μ*g/kg + vehicle olive oil (c) Group C—dexamethasone 120 *μ*g/kg + *α*- tocopherol 60 mg/kg (d) Group D—dexamethasone 240 *μ*g/kg +*α*- tocopherol 60 mg/kg (e) Group E—dexamethasone 120 *μ*g/kg + *γ*-tocotrienol 60 mg/kg (f) Group F—dexamethasone 240 *μ*g/kg + *γ*-tocotrienol 60 mg/kg Dexamethasone was dissolved in olive oils and given intramuscularly daily except on Sunday. Parameters: (a) body composition measurement were done using a dual-energy X-ray absorptiometer for bone mineral density for: (i) left femur and fourth lumbar vertebra (ii) whole body fat mass (iii) whole body lean soft tissue mass (b) bone calcium content.	(a) Bone mineral density Whole body bone mineral density was increased after treatment. However, there was no significant difference between groups at the beginning and at the end of the treatment. (b) Lean soft tissue Whole body lean soft tissue mass was increased after the treatment. There was no significance difference between the groups at the beginning and at the end of the treatment. (c) Whole body fat mass Whole body fat mass was higher in group A compared to before treatment. There was no significant difference between the groups at the beginning of treatment. However, group A, C, and D had higher body fat mass, if compared with group F at the end of treatment. (d) Bone calcium content Fourth lumbar vertebral calcium content was higher in group E and F, if compared with group A and B. No significant difference was seen in left femoral calcium content between groups.	Supplemen-tation with *γ*-tocotrienol was effective in preventing the increase in body fat mass and has the best effect on body composition, while supplementation of *α*-tocopherol was not beneficial at all.

Ahmad et al., 2005 [34]	Animal study Bone histomorphometry analysis Tocotrienol and *α*-tocopherol	32 male Wistar rats (4 weeks old) Rats divided randomly into four groups (8 rats each) Rats were given daily treatment for 8 weeks	First group (control group) was injected intraperitoneally with saline. 2nd group was injected with 2 mg/kg Fe of ferric nitrilotriacetate (FeNTA) which was used to induce diabetes in rats. Group 3 was injected with FeNTA and was given oral doses of 100 mg/kg bodyweight *α*-tocopherol acetate (AT). Group 4 was injected with FeNTA and was given oral doses of 100 mg/kg bodyweight of palm tocotrienol (TT) mixture (*α*-TT 30.7%, *γ*-TT 55.2%, *δ*-TT 14.1%). After 8 weeks of treatment, femurs of the rats were removed for bone histomorphometry measurements. Measurements include: (1) trabecular bone volume (BV/TV) (2) trabecular thickness (TbTh) (3) trabecular number (TbN) (4) mean osteoclast number (OcN) (5) mean osteoblast number (ObN) (6) eroded surface/bone surface (ES/BS) (7) bone formation rate (BFR).	FeNTA injection significantly reduced BV/TV and TbTh of FeNTA and FeNTA + AT groups (*P* < 0.001). TT was able to prevent FeNTA-induced reduction of BV/TV and TbTh. FeNTA + TT group had a higher BV/TV and TbTh, if compared to FeNTA + AT group (*P* < 0.02). Supplementation with TT was able to prevent the increase of ES/BS and prevent the decrease of ObN and ES/BS due to FeNTA administration.	FeNTA generates free radicals which damage bone cells and activate osteoclasts which mimic osteoporotic bone structure. Only palm TT mixture was found to be able to prevent bone damage by FeNTA and is superior to AT in protecting bone against FeNTA toxicity.

Smith et al., 2005 [55]	Animal Study Serum *α*-Tocopherol analysis, serum biochemical marker, oxidative status, bone histology, and bone histomorphometry *α*-Tocopherol	96 Sprague-Dawley rats (8.5 months old) Randomly assigned into 6 groups (16 rats each) Total 13 weeks study period	3 dietary treatments of *α*-tocopherol acetate (AT): (1) low dose (LD)—15 IU/kg (2) adequate dose (AD)—75 IU/kg (3) high dose (HD)—500 IU/kg. AD is the recommended dose and functions as control group. Rats were fed one of 3 diets for 13 weeks. After 9 weeks, rats were either hindlimb unloaded (HU) or maintained ambulatory (AMB) for the final 4 weeks. End of treatment period: (1) whole body dual energy X-ray absorptiometry (DXA) scan was performed (2) blood was taken for serum alkaline phosphatise (ALP) and tartrate resistant acid phosphatise (TRAP) level and activity (3) serum AT level determined using HPLC (4) oxidative status evaluated using plasma ferric-reducing ability (FRAP) and liver thiobarbituric acid reactive substances (TBARS) (5) tibias of the rats were harvested for bone histology and the distal third of femur was harvested for bone histomorphometry. Parameters are as previously described.	FRAP improved with HD treatment group compared to LD and AD groups (*P* < 0.05). Biochemical markers did not result in significant findings. HU group had significantly lower bone histomorphometry results compared to AMB group but AT did not have any effect on bone histomorphometry. Concentrating on HU group, BV/TV increased in AD and HD diets compared to LD diet. (*P* < 0.05). AT helped to maintain TbN during unloading.	*α*-Tocopherol supplementation may provide some protection during hindlimb unloading conditions. However, results were not consistent for all parameters measured.

Chai et al., 2008 [56]	Animal study Bone density assessment of aged osteopenicorchidectomized male rats; control versus high-dose *α*-tocopherol (AT) supplementation *α*-Tocopherol	40 Sprague-Dawley rats (12 months old) Randomly assigned into 4 groups (10 rats each) Total 210 days study period	12-month-old rats were fed AIN-93 M casein-based control diet for 120 days to establish bone loss. Rats were then assigned into 4 groups and given treatment for 90 days: (1) sham operated (Sham) (2) orchidectomized + 75 IU AT (3) orchidectomized + 250 IU AT (4) orchidectomized + 550 IU AT. Analysis: (1) whole body scanning done using DXA at baseline (before surgery), 120 days after surgery and 90 days after dietary treatment. Assessment of bone mineral content (BMC), density (BMD), and area (BMA) (2) assessment of trabecular and cortical bone structures of distal femoral metaphysic and femoral midshaft using Micro-CT. Parameters assessed; BV/TV, TbN, TbTh, structural model index (SMI), connectivity density (Conn.D), cortical bone area (CoArea), thickness (CoTh), porosity (CoP), and medullary area (MArea) (3) measurements of bone biochemical markers; serum osteocalcin, urinary deoxypyridinoline (Dpd), and urinary creatinine concentration.	Mean BMD values of Orx animals were significantly different (lower) compared with sham animals at 120 days (*P* = 0.009) and at 210 days (*P* = 0.001). End of dietary treatment: (1) mean BMD values of AT supplemented groups were not different than Sham group (2) BMC and BMA were not affected with Orx or AT supplementation (3) no significant difference of serum osteocalcin and urinary Dpd noted among the four treatment groups (4) AT supplementation had no effect in preventing Orx-induced unfavourable alterations of trabecular bone parameters (5) AT treatment had no significant effect on CoP, CoTh and CoArea.	Supplement doses of AT do not increase BMD values in male rat model of osteoporosis – unable to reverse bone loss due to gonadal hormone deficiency.

Hermizi et al., 2009 [44]	Animal study Bone histomorphometry Tocotrienol-enhanced fraction *α*-Tocopherol *γ*-Tocotrienol	3-month-old male Sprague-Dawley rats (*n* = 49) Duration: 4-month treatment Rats were randomly assigned to seven groups with seven rats in each group	Group 1 was the baseline. (B) was killed at the commencement of the study. Group 2 and 3 were control (c) and nicotine (N) groups. The C group was treated with normal saline for 4 months and the N group was treated with nicotine for 2 months. The other four groups were nicotine cessation (NC), tocotrienol-enhanced fraction (TEF), GTT, and ATF. Treatment for these groups was performed in two phase. In the first 2 months they were given nicotine (7 mg/kg) and in the following 2 months treatment with vitamin E preparation (60 mg/kg). Rats were sacrificed after 4 months of treatment for bone histomorphometry measurements. Measurement include: (a) structural measurements: (i) trabecular bone volume (BV/TV) (ii) trabecular thickness (Tb.Th) (iii) trabecular number (Tb.N) (b) cellular measurements: (i) osteoclast surface (Oc.S/BS) (ii) eroded surface (ES/BS) (c) dynamic measurements: (i) single-labelled surface/bone surface (sLS/BS) (ii) mineral apposition rate (MAR) (iii) bone formation rate/bone surface (BFR/BS).	All vitamin E treated groups showed significant increase in BV/TV, MAR, and BFS/BS, but there was reduction in sLS/BS and Oc.S/BC compared to the C, N and NC groups. (a) Structural measurements TEF and GTT groups had a significantly higher trabecular thickness, but lower eroded surface (ES/BS) than the C group. (b) Cellular measurements The TEF group had lower ES/BS than the ATF group. (c) Dynamic measurements GTT improved trabecular bone histomorphometric parameter better than TEF and ATF after nicotine administration, by increasing MAR and BFR/BS.	All vitamin E treated group showed significant increase of bone formation and decrease bone resorption. Palm oil tocotrienol mixture was more potent than *α*-tocopherol at reversing the deleterious effects of nicotine on BV/TB and Tb.Th.

Shuid et al., 2010 [57]	Animal study *γ*-Tocotrienol *α*-Tocopherol	3-month-old male Sprague-Dawley rats (*n* = 24) Divided into 3 groups Duration: 4-month treatment	Rats randomly divided into three groups as follows: (a) normal control (NC) Rats were given oral gavage of olive oil (vehicles) (b) *α*-tocopherol (ATF) Rats were given 60 mg/kg ATF body weight orally (c) *γ*-tocotrienol (GTT) Rats were given 60 mg/kg GTT body weight orally. At the end of the treatment rats were killed and both femurs of each rat were dissected free of soft tissue for: (a) bone histomorphometry (b) bone biomechanical test.	The GTT group had significantly higher trabecular bone volume, trabecular number, and trabecular thickness, but significantly lower trabecular separation than ATF group. The GTT group have significantly greater load, higher stiffness, higher stress, higher strain, and higher modulus elasticity, if compared to other group.	Vitamin E supplementation produced greater trabecular volume and number, if compared to control rat. GTT supplementation improves both extrinsic and intrinsic parameters.

Mehat et al., 2010 [58]	Animal study Bone histomorphometry analysis Tocotrienol Tocopherol	3-month-old Sprague-Dawley male rats (*n* = 32) Duration: 4 months Rats were randomly assigned into four groups.	The control group was supplemented with oral gavage vehicle olive oil. The treatment group given orally of 60 mg/kg *α*-tocopherol, *δ*-tocotrienol, and *γ*-tocotrienol. After 4 months of treatment, the rats bone was fluorochrome-labeled with intraperitoneal injection of 20 mg/kg calcein at days 9 and 2 days before the rats were killed. The rats were killed and the left femurs were dissected out and fixed with 70% alcohol. After 1 week the femurs were cut for histology slide samples for: (a) bone static (i) osteoclast number (N.Oc) (ii) osteoblast number (N.Ob) (iii) eroded surface/bone surface ( ES/BS) (iv) osteoid surface/bone surface (OS/BS) (v) osteoid volume/bone volume (OV/BV) (b) bone dynamic (i) single-labelled surface/bone surface (sLS/BS) (ii) mineral apposition rate (MAR) (iii) bone formation rate/bone surface (BFR/BS) (iv) double labelled surface/bone surface (dLS/BS) (v) mineralizing surface/bone surface (MS/BS).	(a) Bone static All vitamin E treated group had significantly higher in N.Ob, OV/BV, and OS/BS but lower N.Oc and ES/BS. GTT group had increased availability of osteoclast for new bone formation (significant increase in N.Ob. OV/BV, and OS/BS). (b) Bone dynamic The percentages of dLS/BS, BFR/BS, MAR, and MS/BS of rat femora are higher in the vitamin E supplemented groups especially in *γ*-tocotrienol group compared to normal control group.	Vitamin E may be able to promote bone growth in rats by increasing trabecular bone volume and osteoid volume but reduce in N.Oc and Es/BS (bone resorption). *γ*-Tocotrienol group demonstrated the best effect in bone static and bone dynamic measurements.

**Table 2:** Characteristics of human studies included in the review.

Study	Type of study/Vit. E analog	Sample/population	Methodology	Results (Vitamin E and osteoporosis only)	Comments or outcomes
Maggio et al., 2003 [59]	Human observational study Vitamin E	1,100 women recruited for instrumental screening for osteoporosis to the Geriatric Division of Perugia University Hospital 150 women (75 osteoporotic and 75 control) gave their consent form and finally enrolled Duration: 12 months Final analysis: 150 women	Study variable included age years, body mass index, self-reported fractures, smoking habits, and other related variables. All pertinent information was collected through a questionnaire that was administrated by trained interviewer. The bone mineral density was measured using dual energy X-ray absorptiometry densitometer. Study subjects underwent a fasting blood withdraw in 20 ml heparin tubes on the day of the bone scan. Blood was kept on ice and centrifuged within 30 min. Plasma aliquot was frozen at −80°C until analysis.	150 women Mean Value: Age: 70.4 ± 8.5 BMI: 25.3 ± 2.9 Years since menopause: 22.8 ± 9 Mean plasma levels of vitamin E was significantly lower in osteoporotic than controls (*P* < 0.001). Antioxidant and MDA plasma level: Plasma vit E (*μ*mol/liter): 46.7 ± 5 Plasma MDA (*μ*mol/liter): 0.34 ± 0.13 MDA did not show any significant difference between osteoporotic and control subjects.	MDA result did not differ between groups. Low antioxidant levels cause antioxidant deficiency and give negative impact on bone mass. Role of vitamin E and osteoporosis needs further investigation.

Macdonald et al., 2004 [60]	Human epidemiological longitudinal study Vitamin E	1064 healthy premenopausal women aged 45–54 who took part in the Aberdeen Prospective Osteoporosis Screening Study. 896 responded to 2nd bone scan and completed questionnaire. 5 excluded (3 women had bisphosphonate therapy, 1 was on wheelchair and another had an outlier dietary calcium intake) Final analysis: 891 women	Participants chosen from women who took part in the Aberdeen Prospective Osteoporosis Screening Study conducted from 1990 to 1993 and had a bone scan done and a completed food-frequency questionnaire (FFQ). This study was a population-based osteoporotic fracture screening program within a 40 km radius of Aberdeen city, Scotland. Between 1997 and 1999, participants were recalled and had a second bone scan done and again completed the FFQ. Inclusion criteria: women who did not have any conditions or taking any medications that might affect their bone metabolism. Anthropometric measurements were taken. Bone mineral density (BMD) measurements of left proximal femur or femoral neck (FN) and lumbar spine (LS) were measured and compared between the first and second measurement. Dietary intake was assessed using the FFQ. The FFQ contained 98 foods or food groups intake of participants recorded over 7 days. Alcohol intake and dietary supplements were also measured. Physical activity level was obtained using the Scottish heart health Study questionnaire.	891 women participants. Mean values: Age (y): 53.9 ±1.6 BMI (kg/m^2^): 26.1 ± 4.4 Total Vitamin E intake (mg): 13.3 ± 32.0 BMD (g/cm^2^): Lumbar spine: 0.998 ± 0.17 Femoral neck: 0.833 ± 0.12 BMD results: No evidence of an association between nutrient intake and BMD change. Vitamin E intake (diet only, not including supplements) was negatively correlated with change in both BMD measurements (*P* < 0.01). Total Vitamin E intake was positively correlated with both BMD measurements but was not significant. Regression analysis: Vitamin E (diet only, not total) accounts for 0.4% of change on femoral neck BMD (*P* = 0.018).	Dietary vitamin E appeared to be a negative predictor for FN BMD change. The authors postulated that this may be due to vit E being a surrogate marker for polyunsaturated fatty acids (PUFA). PUFA anditamin E are highly correlated with PUFA having a negative correlation with FN BMD (*P* < 0.01).

Wolf et al.,2005 [61]	Human epidemiological cross-sectional study Vitamin E	11,393 women aged 50–79 yrs were recruited between 1993 and 1997 to participate in the Women's Health Initiative (WHI) observational study and clinical trial at 3 clinics. BMD was measured and antioxidant intakes were estimated using self-reported food-frequency questionnaire. Women taking oral glucocorticoids, bisphosphonates, calcitonin or tamoxifen were excluded. Final analysis: 11,068 women.	All participants underwent the following data collection. Questionnaire data: (1) demographic data (2) smoking status (3) alcohol intake (4) medication history (5) use of hormone therapy (6) frequency, duration and intensity (strenuous, moderate and mild) of physical activity. (7) diet intake. Nutrients were calculated using Minnesota Nutrition Coding Center database (8) dietary supplements Clinical measurements: (1) weight and height. body mass index (BMI) (2) BMD measurement using dual X-ray absorptiometry; total body, lumbar spine, total hip, femoral neck, and trochanter. Blood measurements: (1) serum antioxidant concentrations; retinol, *α* and *β* carotene, *α* and *γ*-tocopherol, *β*-cryptoxanthin, lycopene, lutein, and zeaxanthin. (2) total cholesterol and triacylglycerols.	11,068 participants, 4.8% had osteoporosis Mean values: Age (y): 63.2 BMI (kg/m^2^): 28.3 ± 5.9 Diet vitamin E intake (mg): 7.8 ± 3.8 Total vitamin E intake (mg): 28.9 ± 49.4 Serum total tocopherols (*μ*g/mL): 18.0 ± 6.0 BMD (g/cm^2^): Whole body: 1.0 ± 0.1 Lumbar spine: 1.0 ± 0.2 Total hip: 0.9 ± 0.1 Femoral neck: 0.7 ± 0.1 Hip trochanter 0.6 ± 0.1 BMD results: Age-adjusted regression analysis resulted in positive association for dietary vitamin E and femoral neck BMD (*P* = 0.002), but negative association for total vitamin E (*P* < 0.0001). However, results after adjusting for multiple important BMD-related covariates (age, BMI, waist circumference, race, education, income, physical activity, etc.) showed no significant association at any BMD sites with vitamin E. No significant association for serum concentration of tocopherols with any BMD sites.	Authors concluded no significant association between vitamin E and BMD. Authors noted that most participants had normal range BMD which may influence association between low BMD and antioxidants.
